# Intramyocardial Transplantation of Undifferentiated Rat Induced Pluripotent Stem Cells Causes Tumorigenesis in the Heart

**DOI:** 10.1371/journal.pone.0019012

**Published:** 2011-04-28

**Authors:** Yuzhen Zhang, Dan Wang, Minglong Chen, Bing Yang, Fengxiang Zhang, Kejiang Cao

**Affiliations:** Department of Cardiology, The First Affiliated Hospital of Nanjing Medical University, Nanjing, China; University of Bristol, United Kingdom

## Abstract

**Background:**

Induced pluripotent stem cells (iPSCs) are a novel candidate for use in cardiac stem cell therapy. However, their intrinsic tumorigenicity requires further investigation prior to use in a clinical setting. In this study we investigated whether undifferentiated iPSCs are tumorigenic after intramyocardial transplantation into immunocompetent allogeneic recipients.

**Methodology/Principal Findings:**

We transplanted 2×10^4^, 2×10^5^, or 2×10^6^ cells from the established rat iPSC line M13 intramyocardially into intact or infarcted hearts of immunocompetent allogeneic rats. Transplant duration was 2, 4, or 6 weeks. Histological examination with hematoxylin-eosin staining confirmed that undifferentiated rat iPSCs could generate heterogeneous tumors in both intracardiac and extracardiac sites. Furthermore, tumor incidence was independent of cell dose, transplant duration, and the presence or absence of myocardial infarction.

**Conclusions/Significance:**

Our study demonstrates that allogeneic iPSC transplantation in the heart will likely result in in situ tumorigenesis, and that cells leaked from the beating heart are a potential source of tumor spread, underscoring the importance of evaluating the safety of future iPSC therapy for cardiac disease.

## Introduction

Induced pluripotent stem cells (iPSCs), first generated four years ago from somatic cells via epigenetic reprogramming [1], offer renewed hope for cardiac stem cell therapy. Previous studies have shown that functional cardiomyocytes could be derived from iPSCs in vitro [2], [3]. Of note, one study reported that intramyocardial delivery of undifferentiated mouse iPSCs within infarcted mouse hearts achieved in situ regeneration of tumor-free cardiac, smooth muscle and endothelial tissue in immunocompetent recipients [4].

However, safety issues should be carefully evaluated prior to clinical application. iPSCs are intrinsically tumorigenic [5]. It is one of the criteria for pluripotency that iPSCs form teratoma in immunocompromised recipients, typically after subcutaneous, intramuscular or intratesticular transplantation. And there have been studies reporting that undifferentiated or predifferentiated iPSCs could also form teratomas in the heart [4] or nervous system [6]-[8] of immunocompromised recipients. Whereas whether the undifferenciated iPSCs will cause tumorigenesis in immunocompetent recipetent is still unknown, and no study has yet been designed to evaluate the tumorigenicity of iPSCs in the cardiac environment. Here, for the first time, we investigated the tumorigenic potential of iPSCs after intramyocardial transplantation into immunocompetent allogeneic recipients and the possible influence of cell dose, transplant duration, and the presence or absence of myocardial infarction.

## Results

**Figure 1:**
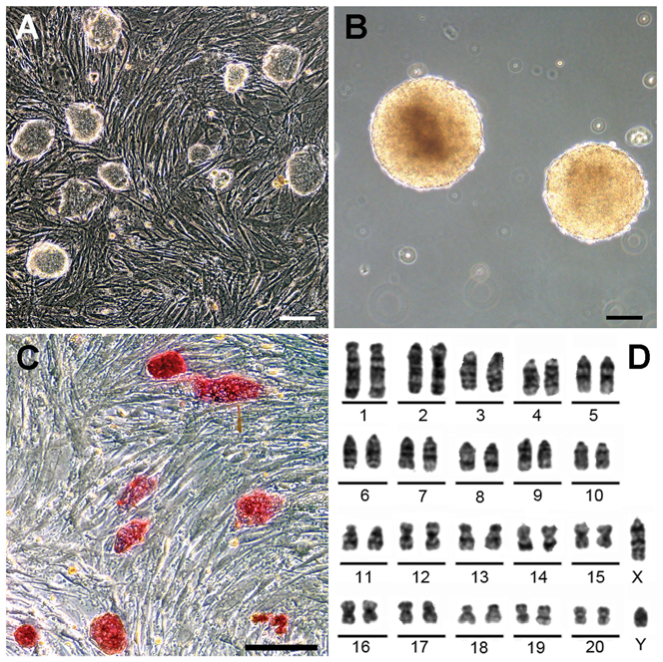
Verification of M13 rat iPSC characteristics. **A,** Mouse embryonic stem cell-like colonies of M13 cells on a feeder layer. **B,** Embryoid bodies on day 6 of a floating culture of M13 cells. **C,** Alkaline phosphatase staining of M13 colonies. **D,** M13 cells maintain a normal diploid karyotype of male rat origen (42XY) at passage 31. **Scale bars:** 100 µm.

We found that the M13 rat iPSC line maintained a stable phenotype and karyotype throughout our experiments (Fig. 1).

**Figure 2:**
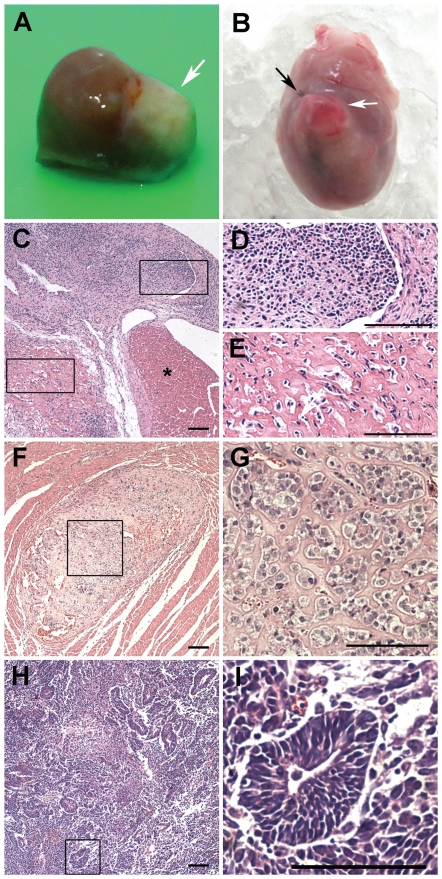
Transplantation of undifferentiated rat iPSCs causes tumorigenesis. **A and B,** Gross pathological evaluation revealed visible tumors **(white arrows)** extending from the left ventricular wall of the heart harvested 2 **(A)** or 6 **(B)** weeks after transplantation. The ligature site is indicated **(black arrow)**. **C**-**I,** Representative series of hematoxylin-eosin-stained sections from harvested tumors. **C,** Heart section from a myocardial infracted (MI) rat sacrificed 2 weeks after cell transplantation showed an undifferentiated cell graft, magnified in **D**, and osteogenic structures (mesoderm), magnified in **E**, in addition to normal cardiac tissue (*). **F,** A clear-edged cartilaginous structure (mesoderm) within the cardiac tissue from an intact rat sacrificed 4 weeks after cell transplantation. **G,** High magnification of the inset in F. **H,** Neural rosettes (ectoderm) in a thoracic cavity tumor of an MI rat sacrificed 4 weeks after cell transplantation. A boxed rosette is magnified in **I**. **Scale bars:** 100 µm.

**Figure 3:**
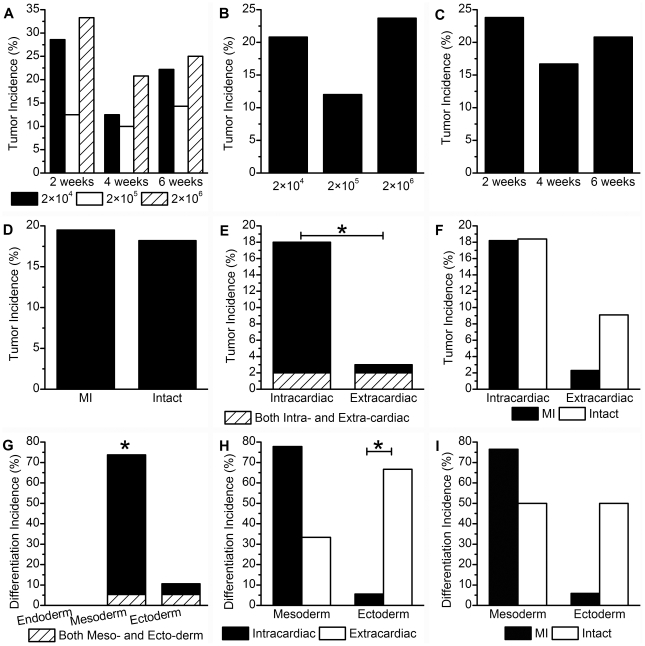
Tumor incidence in rats after transplantation of undifferentiated rat iPSCs and differentiation of the tumors. **A**-**C,** Tumor incidence in MI rats receiving different cell doses and/or subjected to various transplant durations. Cell dosage had no significant influence on tumor incidence, regardless of transplant duration **(A and B)**. Likewise, transplant duration had no significant influence on tumor incidence, regardless of cell dose **(A and C)**. **D,** There was no significant difference in tumor incidence between MI and intact rats. **E,** Intracardiac tumor incidence was significantly higher than that of extracardiac tissues (*P* = 0.001). **F**, Infarction had no significant influence on intracardiac/extracardiac tumor incidence. **G**, The incidence of mesodermal differentiation was significantly higher than endodermal or ectodermal differentiation of tumors (*P*<0.001). **H,** Intracardiac tumors had a lower incidence of ectodermal differentiation than extracardiac tumors (*P* = 0.041), but no significant difference in the incidence of mesodermal differentiation was found between intracardiac and extracardiac tumors. **I,** Infarction had no significant influence on tumor differentiation.

Autopsy results revealed intracardiac and/or extracardiac tumors in 19 out of 98 surviving Sprague-Dawley (SD) rats that received iPSC transplants (details in Table 1). Typically, visible intracardiac tumors extended from the left ventricular wall (Fig. 2A, B), while extracardiac tumors spread in the left thoracic cavity. No tumorigenesis was found in tissues collected from lung, liver, spleen and kidney after histological examination. Intracardiac tumor incidence was significantly higher than that of extracardiac tissues (*P* = 0.001, Fig. 3E).

**Table 1:** Group Information and Tumor Formation.

Model	Transplant	Dose (cells/90 µl)	Duration (wk)	Total Rat Count	SurvivorCount	Tumor-bearing Rat Count
sham	no	no	4	10	10	0
MI	vehicle	0	4	10	6	0
MI	MEFs	2×10^4^	4	10	5	0
MI	riPSCs	2×10^4^	2	10	7	2
MI	riPSCs	2×10^4^	4	10	8	1
MI	riPSCs	2×10^4^	6	10	9	2
MI	riPSCs	2×10^5^	2	10	8	1
MI	riPSCs	2×10^5^	4	10	10	1
MI	riPSCs	2×10^5^	6	10	7	1
MI	riPSCs	2×10^6^	2	10	6	2
MI	riPSCs	2×10^6^	4	30	24	5
MI	riPSCs	2×10^6^	6	10	8	2
intact	riPSCs	2×10^6^	4	11	11	2

MI  =  myocardial infarction; MEFs  =  irradiated mouse embryonic fibroblast cells; riPSCs  =  undifferentiated rat iPSCs.

Hematoxylin-eosin-stained sections from harvested tumors revealed cells that had differentiated to osteogenic (Fig. 2C, E), cartilaginous (Fig. 2F, G), or neural lineages (Fig. 2H, I) among the undifferentiated cells (Fig. 2C, D). Therefore, with no endodermal differentiation observed, these tumors could not be termed “teratomas” or “teratocarcinomas”. Furthermore, the incidence of mesodermal differentiation was significantly higher than that of endodermal and ectodermal differentiation (*P*<0.001, Fig. 3G).

To determine if tumorigenesis was exacerbated by the dosage of transplanted cells, 2×10^4^, 2×10^5^, or 2×10^6^ iPSCs were delivered intramyocardially into myocardial infarcted (MI) rats. However, no significant difference in tumor incidence was found among these conditions (Fig. 3A, B). This suggests that the tumorigenesis observed in MI rats was dose independent within the numbers of cells used for this experiment.

To evaluate the timecourse of tumorigenesis and differentiation, we examined the engrafted cells 2, 4 or 6 weeks after transplantation. We found no significant difference in tumor incidence among time points (Fig. 3A, C). In addition, undifferentiated cells were found at every time point examined.

To assess the possible influence of the host microenvironment on in vivo differentiation of transplanted cells, we compared the incidence of mesodermal and ectodermal differentiation between intracardiac and extracardiac tumors, as well as MI versus intact rats. Intracardiac tumors had a lower incidence of ectodermal differentiation than extracardiac tumors (*P* = 0.041), but no significant difference in the incidence of mesodermal differentiation was observed between intracardiac and extracardiac tumors (Fig. 3H). In addition, there was no significant difference in mesodermal or ectodermal differentiation between tumors generated in MI and intact myocardium (Fig. 3I). Furthermore, the cardiac host microenvironment (MI versus intact myocardium) had no significant effect on either total tumor incidence (Fig. 3D) or intracardiac/extracardiac tumor incidence (Fig. 3F). Apart from a slightly lower tendency toward ectodermal differentiation in the myocardium compared to the thoracic cavity, these results suggest that the host microenvironment had little influence on tumor incidence and differentiation.

## Discussion

In this report, we show that undifferentiated rat iPSCs can generate heterogeneous tumors in intracardiac and extracardiac sites after intramyocardial delivery in immunocompetent allogeneic rats. In addition, tumors formed in these rats regardless of cell dose, transplant duration, or the presence or absence of MI.

In contrast to our results, previous work reported that intramyocardial delivery of undifferentiated mouse iPSCs, generated by lentivirus-mediated transduction with Oct4, Sox2, c-Myc and Klf4, resulted in regeneration of cardiac, smooth muscle and endothelial tissue without tumorigenesis in immunocompetent mice 8 weeks after transplantation [4]. Also, embryonic stem cell (ESC) studies have suggested that the cardiac microenvironment might provide paracrine signals to direct in vivo differentiation of ESCs into cardiomyocytes within 2 to 8 weeks [9]-[11]. However, additional studies found no guided differentiation of ESCs in the heart. Rather, teratoma formation was observed between 3 and 4 weeks after transplantation [12]-[14].

Here we investigated whether iPSCs are more likely to undergo guided differentiation in infarcted myocardium, which is likely to release more paracrine factors, or intact myocardium, which is considered as a less hostile microenvironment for stem cells to survive. We observed that neither the infarcted nor intact cardiac microenvironments could guide iPSCs toward the cardiac lineage. Rather, heart tissue was permissive for the generation of tumors consisting of undifferentiated as well as lineage-committed cells, thus challenging the hypothesis of “host microenvironment guided differentiation” of iPSCs.

We found no significant difference in tumor incidence among the three doses of iPSCs tested. By contrast, some ESC transplantation studies showed that tumor incidence was positively correlated with the transplanted cell dose [13]-[15]. The results of ESC studies may be explained that: 1) paracrine signals released by the surrounding tissue might not permeate into the core of a large cell graft, leaving cells to differentiate randomly; and 2) autocrine factors released by pluripotent cells might accumulate and trigger tumorigenesis in larger cell grafts. However, no enhancement of cardiac differentiation has been discerned at the margin versus the center of an intracardiac teratoma generated from mESCs [13], in spite of an assumed better paracrine signal permeation at the graft-host border. Moreover, teratomas have been observed in transplants of as few as 2 ESCs [16], which is presumably not a high enough cell number to accumulate autocrine factors. Furthermore, the cell dose threshold for tumorigenesis has been reported to range from 2 to hundreds of thousands of ESCs [13]-[16], suggesting that experimental variation is a major factor in tumorigenesis. Therefore, a safe cell dose threshold may not be attainable presently for a given transplantation setting, and more caution is appropriate.

The extracardiac tumors were found in the left thoracic cavities, leaving organs with a typically high metastatic risk free of tumor. Thus we postulate that these tumors originated from cells leaked into the thoracic cavity during or after transplantation, rather than from migrating cells. Hence, cell transplantation techniques should be refined to improve cell retention, not only to enhance transplant efficiency, but also to diminish the danger of tumor spread.

No graft or tumor was evidenced in 80.6% of the surviving recipients. There are 3 possible reasons. First, after injection into beating myocardium, mechanical leakage and washout may account for a major portion of cell loss after cell implantation [17]. If these leaked cells retained in the thoracic cavities, they could form extracardiac tumors as we observed in 3 cases. If the cells completely washed out, then no tumor would be generated. Second, grafts may be rejected by the recipients’ immune system. The M13 iPSCs, originated from SD outbred rat, and feeder cells of mouse origin remained in the prepared cell suspension, as allografts and xenografts respectively, may induce rejection in recipient SD rats. Third, grafts may be too small to be distinguished in gross examination and fail to be sampled for microscopic examination.

Although the underlying mechanisms for the tumorigenesis observed in this study are not yet understood, there are several possibilities. First, oncogene involvement and genome integration might play a role, because the M13 rat iPSC line was induced using integrating lentiviral vectors to deliver the four Yamanaka factors, including the oncogene c-Myc. In such a scenario, insertional mutagenesis and transgene reactivation can occur. Besides, culture adaptation may have occurred because, although these cells maintained a stable phenotype and karyotype in culture, they may have undergone oncogenic transformation caused by undetected genetic or epigenetic alterations. Furthermore, recent studies have shown that iPSCs are not completely reprogrammed to a ground state of pluripotency [18]-[20] and still retain some epigenetic memory of their former fate, preferring to differentiate along lineages related to donor cell identity [21]-[23]. For example, M13 iPSCs were induced from bone marrow cells, which may explain the prevalence of chondro-osteogenic differentiation observed in this study, in spite that M13 cells support the differentiation into cell types of all three germ layers after intramuscular injection into immunocompromised recipients [24].

Some most recent studies have reported that the reprogramming process and subsequent culture of iPSCs in vitro can induce genetic and epigenetic abnormalities in these cells, regardless of the reprograming methods, integrating or non-integrating [25]-[29]. Potential risks exist in all stages of the induction of iPSCs and their subsequent expansion. So iPSCs generated by any method should be subjected to rigorous and procedural examination to confirm their safety prior to clinical application.

In conclusion, our study demonstrates that, allogeneic iPSC transplantation into the heart can cause in situ tumorigenesis in immunocompetent recipients, and that the cells leaked from the beating heart likely serve as a source of tumor spread. These results imply that, for iPSC-based cardiac regeneration therapy, the safety issue should be carefully addressed, and that the elimination of undifferentiated cells from the grafts is necessary not only in immunocompromised recipients but also in immunocompetent recipients. Therefore, further studies are warranted to thoroughly examine the possible mechanisms of iPSC tumorigenicity, and to refine the techniques of directed differentiation and purification of iPSC derived cardiomyocytes.

## Materials and Methods

### Ethics Statement

This study was carried out in strict accordance with the recommendations in the Guide for the Care and Use of Laboratory Animals of the National Institutes of Health. The protocol was approved by the Experimental Animal Ethics Committee of Nanjing Medical University (20100219), China. All surgery was performed under pentobarbital anesthesia, and all efforts were made to minimize suffering.

### Cell Culture and Characterization

The M13 rat iPSC line, which was generated from adult SD rat bone marrow cells by lentivirus-mediated transduction with Oct4, Sox2, c-Myc and Klf4 [24], was kindly provided by Dr Lei Xiao (Shanghai Institutes for Biological Sciences, Chinese Academy of Sciences, Shanghai, China).

Rat iPSCs were maintained on irradiated mouse embryonic fibroblast cells (MEFs, SiDanSai) in Knockout Dulbecco's modified Eagle's medium (DMEM) supplemented with 10% ESC grade fetal bovine serum, 10% KnockOut serum replacement, 0.1 mM non-essential amino acids, 1 mM L-glutamine, and 0.1 mM ß-mercaptoethanol (all from Invitrogen) [24]. Rat iPSCs were dissociated into single cells with TrypLE (Invitrogen) and split at a ratio of 1∶5-1∶15 every 2-3 days.

To form embryoid bodies, rat iPSCs were dissociated into single cells and transferred to a Petri dish in differentiation medium consisting of DMEM (Invitrogen) supplemented with 10% fetal bovine serum (Thermo scientific) [24].

Alkaline phosphatase staining was performed 2 days after cell passaging using an Alkaline Phosphatase Detection Kit (Millipore) according to the manufacturer's instructions.

Karyotyping was performed at the Xiangtan Center Hospital using standard protocols for high-resolution G-banding. More than 20 metaphase nuclei were examined.

### Cell Preparation for Transplantation

M13 iPSCs at passage 22 to 24 were prepared for transplantation. On transplantation day, cells in culture were enzymatically dissociated into a single cell suspension containing undifferentiated rat iPSCs and a considerable portion of MEFs. The mixed cell suspension was plated into a culture flask and incubated for 15 minutes to allow most of the MEFs to attach, leaving mainly rat iPSCs suspended. Undifferentiated rat iPSCs were then collected, washed and re-suspended in Knockout DMEM. A MEF suspension in Knockout DMEM was also prepared for transplantation to provide a control for the possible confounding factor of residual MEFs in the rat iPSC suspension. Harvested cells were kept on ice for optimal viability prior to transplantation.

### Rat Model of Myocardial Infarction and Cell Transplantation

SD rats were housed under controlled conditions in a 12-hour light/12-hour dark cycle at the Experimental Animal Center of Nanjing Medical University, and were provided standard rodent chow and water ad libitum.

A total of 151 female SD rats (6- to 10-wk-old, 200 to 250 g) were subjected to the transplantation protocols summarized in Table 1.

Specifically, rats were anaesthetized by intraperitoneal injection of pentobarbital (50 mg/kg body weight, Roche). After endotracheal intubation and initiation of ventilation (room air, rate 60 cycles/min, tidal volume 1 ml per 100 g of body weight), the heart was exposed by left intercostal thoracotomy via a lateral incision between the fourth and fifth ribs and the pericardium removed to access the left anterior descending artery and its branch. Under direct visualization, an 8-0 nylon atraumatic suture was passed through the epicardial layer around the origin of the major branch(es) and several minor branches of the left coronary artery [30]. The ligatures were tied to occlude the coronary arteries, while in sham-operated rats, the ligatures remained loose. Myocardial ischemia was confirmed by ECG and color change of the left ventricular wall.

Within 10 minutes after ligation, MI rats received intramyocardial injections to the peri-infarct region at 3 different sites (30 µl per site), using a 29-gauge needle attached to an insulin syringe; while sham operated rats received no injection. The chest was closed layer by layer with a 3-0 suture and rats were allowed to recover under care.

### Histological Study

After completion of the various survival periods, surviving rats were euthanized with an overdose of pentobarbital (Roche). Hearts were rapidly excised and autopsies were performed to search for possible tumor formation in thoracic cavities, lungs, livers, spleens and kidneys, and suspicious tissues were collected. Explanted organs and tissues were fixed with 4% paraformadehyde for 24 hours (some of the heart specimens were frozen in liquid nitrogen prior to fixation), then embedded in paraffin, sectioned at 4 µm thickness and stained with haematoxylin-eosin. Slides were evaluated by 2 independent pathologists who were blinded to the study.

### Statistics

Percentages were compared by chi-square test or Fisher's exact test. *P*<0.05 was predetermined as significant.
